# Effectiveness of containment strategies and local cognition to control vehicular traffic volume in Dhaka, Bangladesh during COVID-19 pandemic: Use of Google Map based real-time traffic data

**DOI:** 10.1371/journal.pone.0252228

**Published:** 2021-05-27

**Authors:** Niaz Mahmud Zafri, Sadia Afroj, Mohammad Ashraf Ali, Md. Musleh Uddin Hasan, Md. Hamidur Rahman

**Affiliations:** 1 Department of Urban and Regional Planning, Bangladesh University of Engineering and Technology (BUET), Dhaka, Bangladesh; 2 Asian Disaster Preparedness Center (ADPC), Dhaka, Bangladesh; Tsinghua University, CHINA

## Abstract

**Background:**

To prevent the viral transmission from higher infected to lower infected area, controlling the vehicular traffic, consequently public movement on roads is crucial. Containment strategies and local cognition regarding pandemic might be helpful to control vehicular movement. This study aimed to ascertain the effectiveness of containment strategies and local cognition for controlling traffic volume during COVID-19 pandemic in Dhaka, Bangladesh.

**Method:**

Six containment strategies were considered to explore their influence on traffic condition, including declaration of general holiday, closure of educational institution, deployment of force, restriction on religious gathering, closure of commercial activities, and closure of garments factories. Newspaper coverage and public concern about COVID-19 were considered as local cognition in this research. The month of Ramadan as a potential event was also taken into account considering it might have an impact on the overall situation. Average daily journey speed (*ADJS*) was calculated from real-time traffic data of Google Map to understand the vehicular traffic scenario of Dhaka. A multiple linear regression method was developed to comprehend the findings.

**Results:**

The results showed that among the containment strategies, declaration of general holiday and closure of educational institutions could increase the *ADJS* significantly, thereby referring to less traffic movement. Besides, local cognition could not significantly affect the traffic condition, although the month of Ramadan could increase the *ADJS* significantly.

**Conclusion:**

It is expected that these findings would provide new insights into decision-making and help to take appropriate strategies to tackle the future pandemic situation.

## Introduction

**Fig 1:**
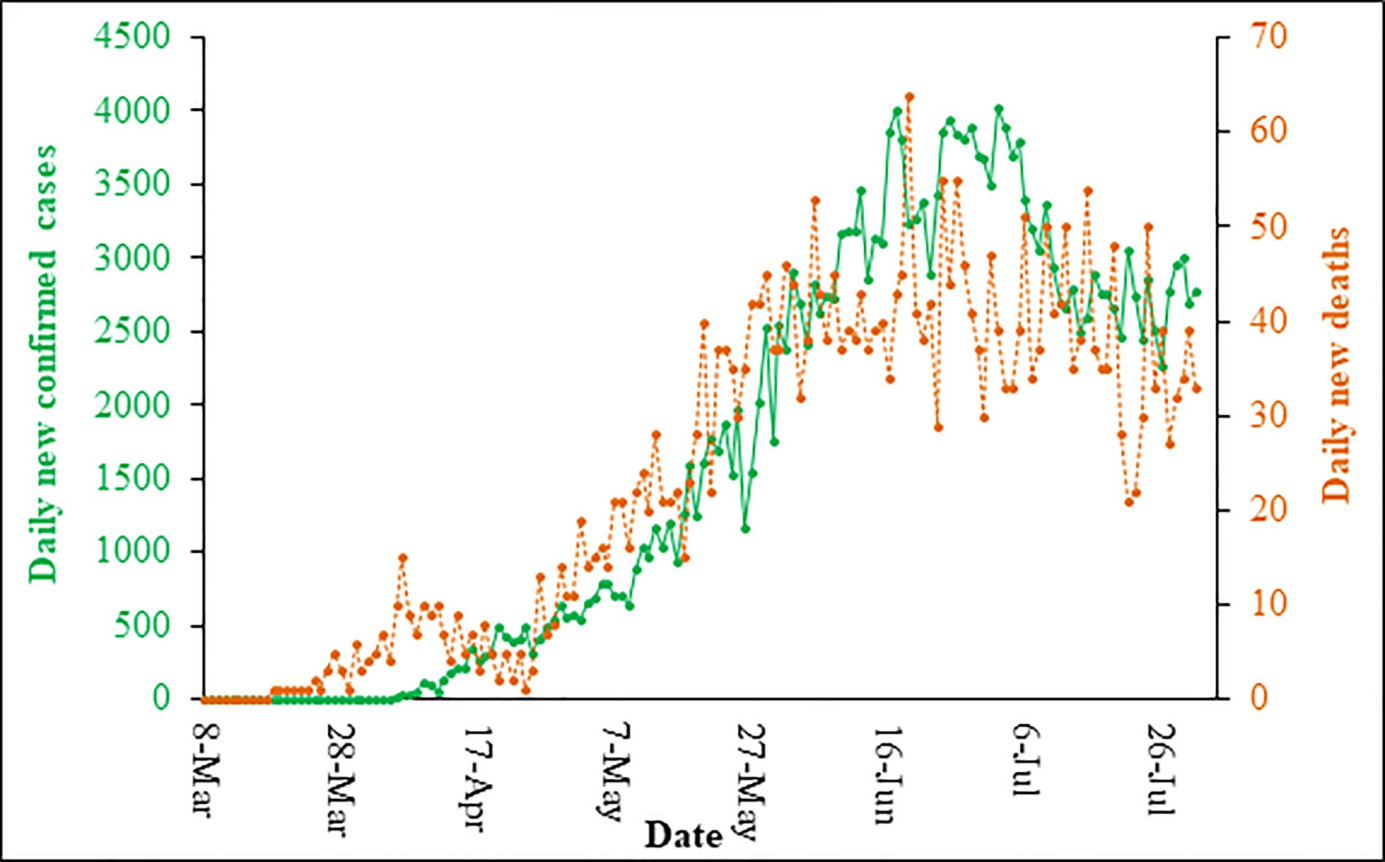
Nationwide confirmed cases and deaths in Bangladesh [22].

The Novel Coronavirus (SARS-COV-2) had an explosive outbreak after its first reporting on December 2019 in Wuhan, Hubei province, China [1]. This virus generally transmits through human-to-human interaction and can survive on the exposed surfaces [2, 3]. In March 2020, the World Health Organization (WHO) declared the epidemic of this Coronavirus Disease 2019 (COVID-19) as a global pandemic, and until now, 213 countries and territories around the world have reported the spread of COVID-19 [4]. Bangladesh has become one of the top 20 countries containing the highest COVID-19 confirmed cases with a rapid escalation of spread after its first identification on March 08, 2020 [5]. As of November 15, 2020, 0.43 million confirmed cases and 6,173 deaths have been reported in Bangladesh and the incidences are showing a progressive trend over time (**Fig 1**). Dhaka, the epicenter of the domestic outbreak, has evidenced 0.125 million confirmed cases, accounting for around 30% of country’s total confirmed cases [5].

With human mobility, the transmission risk of virus is growing over space. To prevent the exponential growth of viral spread, a large number of public health interventions have already been implemented worldwide considering global and local contexts [6, 7]. Several behavioral risk-reduction strategies, including compulsory use of masks, frequent hand-washing, avoidance of individual interactions, and maintenance of basic hygiene are mandated to mitigate the individual risk [8, 9]. Both pharmaceutical and non-pharmaceutical countermeasures are resorted to alleviate societal risk and prevent the spread of COVID-19. The measures include complete lockdown of cities, social-distancing, restrictions of traffic movements, prohibitions of international traveling, mandatory quarantine of international travelers, isolation of suspected and confirmed cases, increase of testing capacity, and responsive use of drugs [8, 10, 11]. Maintaining social distance with restricted movements and avoiding physical contact can reduce the risk of spread significantly, which has already been proven from the transmission model [12].

Besides the aforementioned initiatives, some other interventions have also been embedded considering the local demand to reduce exposure. Curbing the educational and business operations, closure of non-essential businesses and factories, shutting the markets and shopping mall exception for essential needs, maintaining sanitary cordon, and restriction on large scale social gathering are certain containment strategies imposed by several state governments [8, 11, 13]. All of these strategies have been imposed for the reduction of vehicular traffic to keep people stay-at-home [14, 15]. The Government of Bangladesh (GoB) has also enforced some of these measures, even though the methods of enactment differ notably considering the local context. Declaration of general holiday, closure of educational institutions and garments factories, deployment of forces, restriction on the religious and social gathering, shutting down the shopping centers, and prohibition of inter-district movements as well as public transport are some of the containment strategies adopted in Bangladesh [16, 17]. Usually, the exposure is greater if people spend a longer time in a viral loaded space or public gathering. The potential risk of transmission also increases if vehicles ply on the roads frequently. The more traffic on the roads, the more people exposed to the virus and the more likelihood of viral spread from the higher infected to the lower infected area [13, 18]. To keep people staying at home and reduce the vehicular movement on roads for limiting the transmission, these containment strategies were taken in Bangladesh.

In addition to these interventions, local cognition about pandemic also helps to tackle the situation by enhancing public awareness that can be reflected through local media coverage and public interest towards COVID-19. Being the most noteworthy pandemic, the media coverage on COVID-19 has been notably higher. In this regard, local newspaper acts as an elevated platform by highlighting the situation updates and intervention strategies to prevent the impact of the outbreak which was already evidenced during Influenza and Swine Flu pandemic [19, 20]. Generally, the knowledge of epidemics is not easily perceivable to people. Local newspaper presents the information time-to-time in an understandable way and keeps people updated and aware about the circumstances. Moreover, people are inherently curious about the pandemic and new normal situation. From their own interest, they try to gather more knowledge through searching on COVID-19 at newspapers and web sources so that they can be informed about how to mitigate the individual risk [21]. Thus, local media coverage and public concern towards COVID-19 can increase their awareness level by making them cognizant and shaping their decision to go out of home. In this manner, local media and public concern might influence the vehicular movement on roads.

In search for a new healthy sustainable normal, while many dedicated researchers are working in the pharmaceutical arena, many of them are working on non-pharmaceutical fields to find the best countermeasures to assist the decision-makers for tackling the pandemic situation [23]. This study intended to ascertain the effectiveness of different containment strategies and local cognition to control vehicular traffic volume on the roads in Dhaka for preventing the transmission of COVID-19.

## Materials and methods

### Conceptualization

For assessing the effectiveness of different containment strategies and local cognition on vehicular traffic volume reduction, the data of traffic volume on the roads of Dhaka were required. However, collecting the traffic volume data for a large city like Dhaka is practically troublesome as well as costly, and especially becomes perilous during such pandemic situation. Hence, journey speed was envisaged instead of traffic volume as proxy variable. Journey speed indicates the effective speed of the vehicles, which is measured dividing the travel distance between origin and destination by total time taken to finish the journey, including any stoppage, congestion, and other internal delays of trip [24]. The change in number of vehicular traffic on roads has significant impact on journey speed and the intercourse between them was already asserted in literature. According to the basic traffic flow theory, speed-density association on a roadway is linear, having a negative slope, which means that if the density decreases, the speed of the roadway increases and vice versa [25]. Recent studies found that the lower journey speed denotes the traffic comes to a standstill due to congestion and the higher journey speed imparts the scenario of comfortable condition [26–28]. Therefore, the higher journey speed implies lower traffic volume and the lower journey speed is caused by higher traffic volume on roads.

Journey speed data can be determined from Google live traffic map that integrates real-time traffic information. Google generally accumulates the traffic data from various sources like road sensors, car fleets, and mobile users through GPS system. “Google Maps” application on Android and iOS constantly provides real-time traffic data to Google. The provided data from a device are further compared with data received from other devices staying within same area. After that, the traffic data are cross-matched and validated using historical data found from several local transport departments and private data providers [29, 30]. The use of real-time traffic data from “Google Maps” is upturning in the fields of transport geography, accessibility, route optimization, and traffic impact analysis [31–33], and the accuracy of the data had also been substantiated for different spatial and temporal dimensions [34]. This real-time traffic data was also used in this research to apprehend the journey speed.

### Data collection

**Fig 2:**
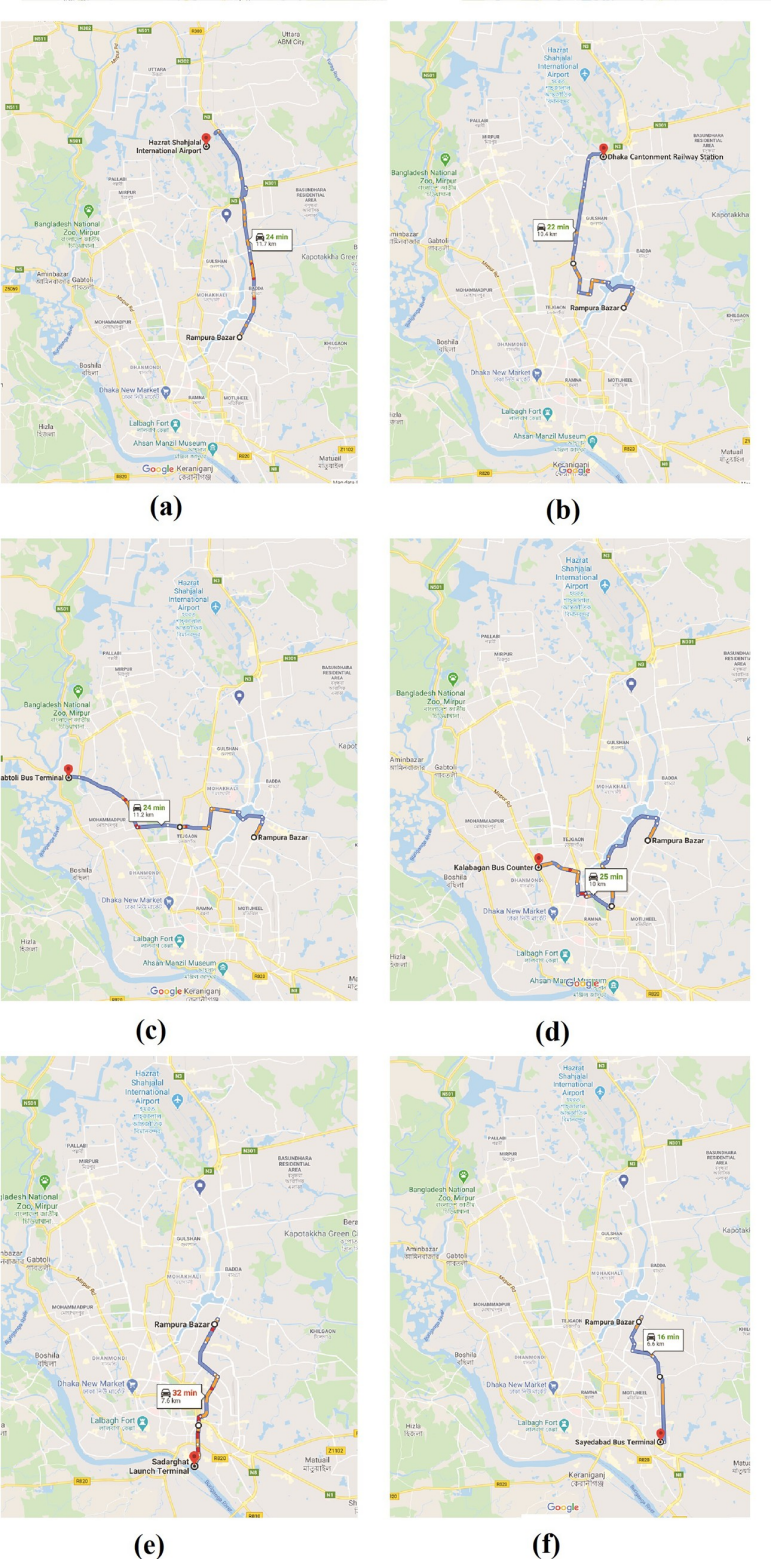
Routes from origin (Rampura Bazar) to destinations [(a) Hazrat Shahjalal International Airport, (b) Dhaka Cantonment Railway Station, (c) Gabtoli Bus Terminal, (d) Kalabagan Bus Counter, (e) Sadarghat Launch Terminal, and (f) Sayedabad Bus Terminal].

To collect the data of journey speed, an origin (Rampura Bazar) and six destinations (Sadarghat Launch Terminal, Dhaka Cantonment Railway Station, Gabtoli Bus Terminal, Hazrat Shahjalal International Airport, Sayedabad Bus Terminal, and Kalabagan Bus Counter) were selected in such a way that the collected data could provide a complete picture of the traffic condition of entire Dhaka. There are several routes available between the origin and each destination. Among them, a route for each destination was chosen (**Fig 2)**. The principle of route choice between origin and destinations was that all selected routes together could represent the traffic scenario of all the important roads of Dhaka. After entering the address of origin and each destination in “Google Map”, the map provided data of travel distance (route-wise) and travel time based on real-time traffic scenario. Journey speed was calculated afterwards dividing travel distance by travel time. For each day, journey speed data were taken at four different periods: morning (9 am- 10 am), noon (1 pm- 2 pm), evening (5 pm- 6 pm), and night (8 pm- 9 pm). Then by averaging all journey speed data of a particular day, average daily journey speed (*ADJS*) was calculated. In this way, *ADJS* data were assembled from the first country’s COVID-19 detection date (March 8, 2020) to July 31, 2020 (145 days in total). The traffic data on weekends and predetermined government holidays- 49 days in total- were excluded from the analysis to assess the effectiveness of the containment strategies and local cognition precisely. Therefore, finally, the study used *ADJS* data for 96 days.

Data related to the containment strategies were accumulated through the regular observation of newspapers, television news, and reports on COVID-19 situation. To operationalize the local cognition based on the learning from the studies of Shilpa, Kumar [20], Wilson, Iannarone [19], and Knipe, Evans [21], we envisaged “the daily number of published articles on COVID-19 on a local newspaper” and “COVID-19 search trend data on Google” as proxy variables. Details about the considered variables are elucidated in **Table 1**. In this study, the *“NEWS”* data were collected from a leading and renowned newspaper of Bangladesh named *bdnews24*.*com*. Even though the “month of Ramadan” variable is not similar to the considered variables, it was also taken into account considering this month has huge influence on the people of Dhaka as 90% of the people of Bangladesh are Muslim [35]. The result of the study might be deviated from the actual scenario if this variable was not considered.

**Table 1:** Containment strategies (independent variables) description.

Variable Acronym	Variable	Variable description	Code or Value	Data Source
*GHLDY*	Declaration of general holiday	The government declares general holiday for all types of public and private administrative, commercial, and industrial activities for a certain period.	0= General holiday 1= Non-holiday	[13, 17, 36, 37]
*EDUCTN*	Closure of educational institutions	All educational institutions remain closed under the announcement of the Ministry of Education.	0= Closed 1= Opened	[38–40]
*FORCE*	Deployment of force	The civil administrations and armed forces are deployed nationwide as per the government directive under “In Aid to Civil Power” to control the transmission of COVID-19.	0= Not in action 1= In action	[17, 41]
*RELIGN*	Restriction on religious gathering	Restriction on all kinds of religious gathering is imposed nationally to prevent the spread of COVID-19.	0= Restricted 1= Permitted	[42, 43]
*MALL*	Closure of market and shopping mall	All markets and shopping malls across the country are declared to be closed except the grocers’ shop, shops of daily essential commodities, and medicine shops.	0= Closed 1= Opened	[13, 44, 45]
*GRMT*	Closures of garments factories	All garments factories of the country are announced to be closed as per the directive of respective authority (BGMEA and BKMEA) for a certain period.	0=Closed 1= Opened	[13, 46]
*PC*	Public concern and interest about COVID-19	Coronavirus search trend in Google in Bangladesh. Measures in the unit of “interest over time”. A value of 100 is the peak popularity for the term. A value of 50 means that the term is half as popular. A score of 0 means the term is not popular at all.	Continuous variable	https://trends.google.com/
*NEWS*	Newspaper coverage on COVID-19	Number of articles published in bdnews24.com newspaper per day on COVID-19 pandemic.	Continuous variable	https://bdnews24.com/
*RMDN*	Month of Ramadan	Days in the period of Ramadan month	0= Days not in Ramadan 1= Days in Ramadan	[47]

### Data analysis

The data analysis was accomplished in two stages: descriptive analysis and two-stage statistical analysis. Initially, descriptive analysis was conducted where a chart was prepared by plotting *ADJS* versus date of the corresponding day. The status of containment strategies and month of Ramadan was also adjoined with the chart to expose the change in average daily journey speed more clearly. Two line charts were also created to show the relationship between *PC* and *NEWS* with *ADJS*. Later on, in the first stage of statistical analysis, the univariate analysis was conducted where Independent Sample t Test was conducted using each of the seven categorical independent variables with the dependent variable individually. Pearson Correlation test conducted in case of continuous independent variables. In the second stage, multivariate analysis was conducted where linear regression method was adopted for the modeling purpose. This method refers to a linear approach to comprise the relationship between a continuous dependent variable and one or more independent variables. Generally, the relationship between the dependent variable and independent variables is explained through the following equation.

$$Y=\alpha +\beta_{1}X_{1}+\beta_{2}X_{2}+\beta_{3}X_{3}+\ldots \ldots .+\beta_{i}X_{i}+\varepsilon$$ Eq (i)

Here, Y is the dependent variable, *α* is the constant, *β*_*i*_ (*i*= 1, 2, 3,….., *n*) is the regression coefficients, *X*_*i*_ (*i*= 1, 2, 3,….., *n*) are the independent variables, *n* is the number of independent variable, and ε is the error term of the model.

For this study, the dependent variable was *ADJS* and independent variables were six containment strategies, *PC*, *NEWS*, and *RMDN* variables. A best fit multiple linear regression model was developed through stepwise forward procedure using all the independent variables with the dependent variable. Variance Inflation Factor (VIF) was used to ensure that the developed model was free from multicollinearity and only the uncorrelated independent variables were present in the model. After developing the model, the assumptions of regression model (e.g., linearity, homoscedasticity, auto-correlation, outlier, normality, and multicollinearity) were checked. Finally, the results of the analyses were reported and discussed simultaneously.

## Result and discussion

**Fig 3:**
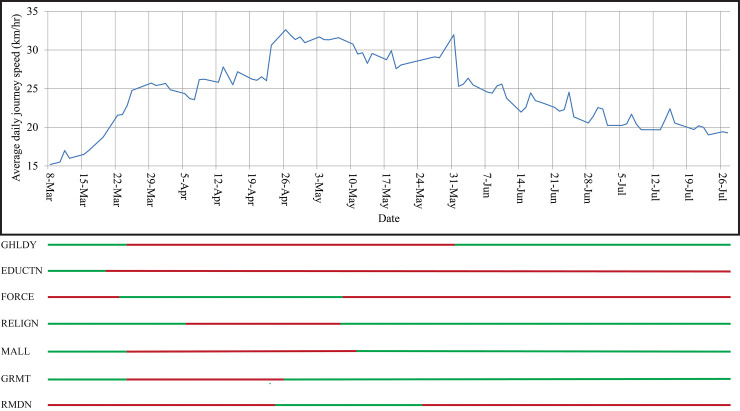
Average daily journey speed (*ADJS)* by date as well as containment strategies and the month of Ramadan. (Here, for the chart portion, blue line is the trend line. Whereas, in the containment strategies portion, red line means variable value= 0 and green line means variable value= 1 according to **Table 1**).

**Fig 4:**
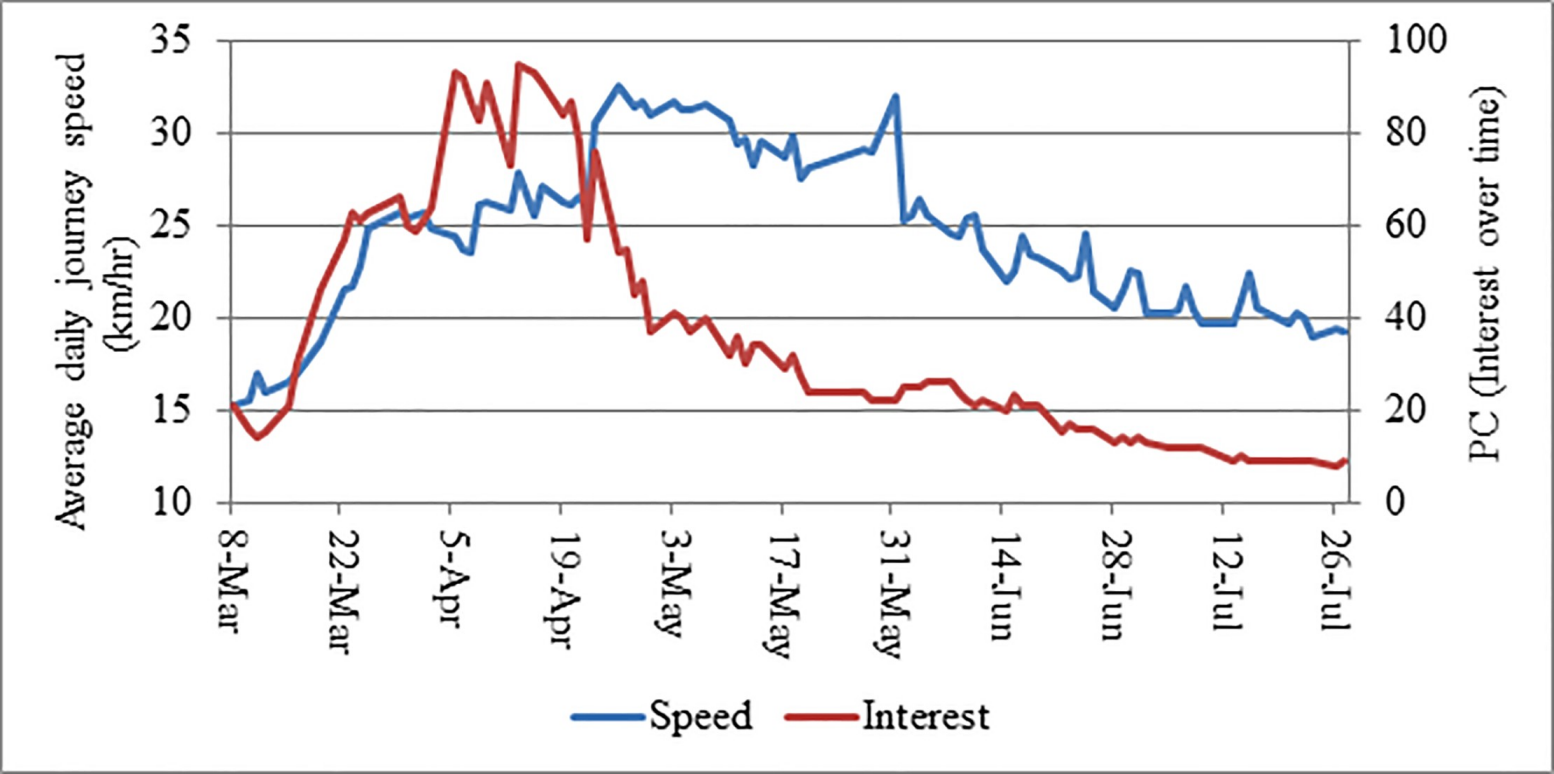
Time series of average daily journey speed (*ADJS)* and public concern and interest about COVID-19 (*PC*).

**Fig 5:**
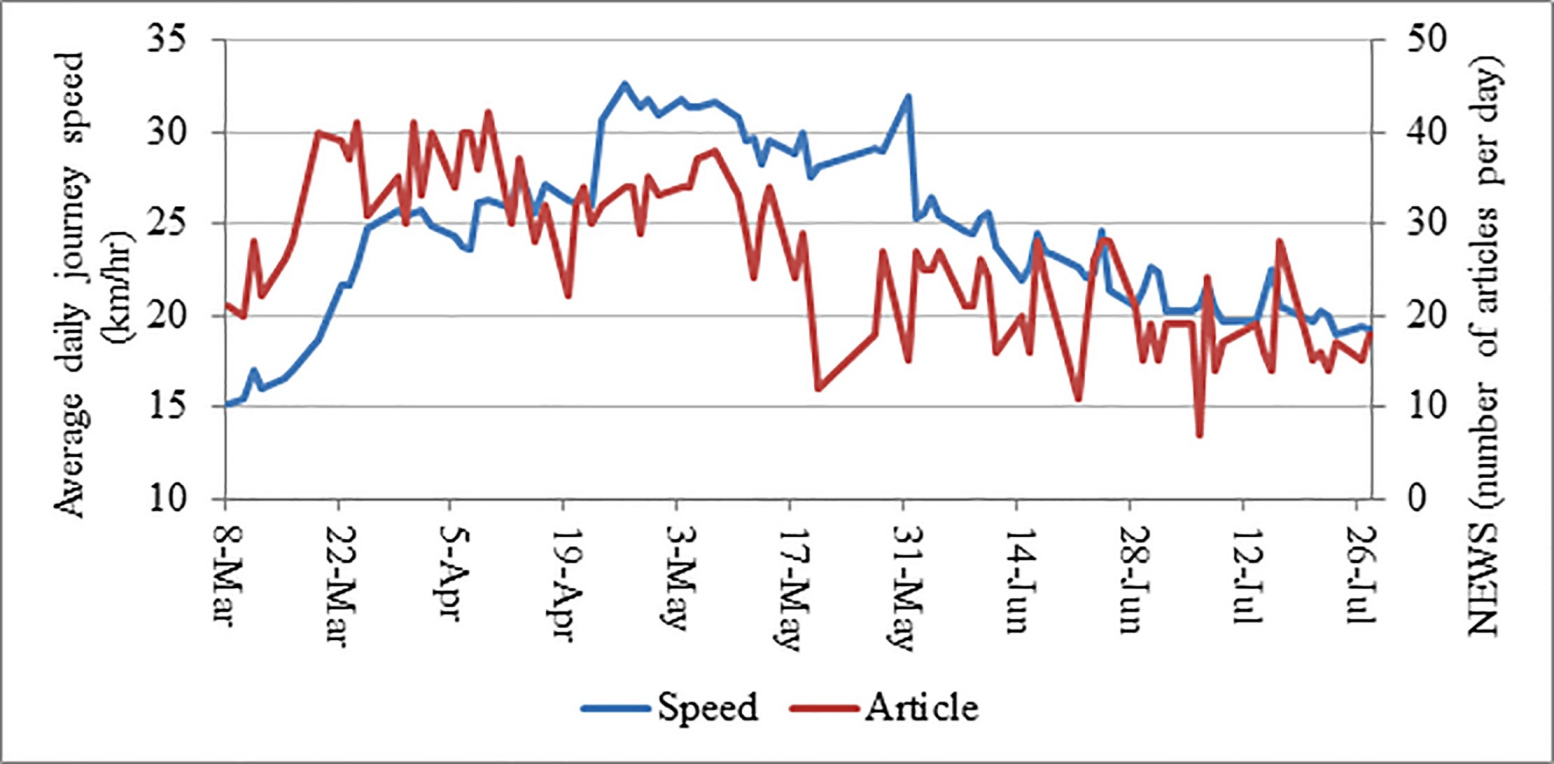
Time series of average daily journey speed (*ADJS)* and newspaper coverage on COVID-19 (*PC*).

The results of the descriptive analysis are exhibited in **Figs 3**–**5**. From **Fig 3**, it is clear that the *ADJS* drastically increased after March 17, 2020. By March 26, most of the containment strategies were in action. The value of *ADJS* gradually increased with time and significantly decreased in the month of June when most of the containment strategies were relaxed. At the end of April, this value was almost twice than the value in the eve of March. Therefore, **Fig 3** illustrates that the containment strategies had influence on *ADJS*, and consequently helped to control vehicular traffic on roads.

The results of the univariate analysis are given in **Table 2**. Afterwards, a best fit multiple linear regression model was developed during multivariate analysis (**Table 3**). Assumptions of the multiple linear regression analysis were fulfilled by the developed model (**S1 Appendix**). There was no evidence of multicollinearity (Variance Inflation Factor (VIF) values < 5). In case of model statistics, *F*-test result showed that the developed multiple regression model was statistically significantly, *F*(4, 85) = 72.506, *p*-value = 0.000 (*p*-value < 0.01). The *R^2^* value (*R^2^*=0.773) of the model was also high. Regression coefficients (*B*), upper bound and lower bound for coefficients (*B*) at 95.0% Confidence Interval, *p*-values, and *VIF* are demonstrated in **Table 3**. This model is presented in the following Eq (ii).

**Table 2:** Results of the univariate analysis (Relationship between dependent variable and each of the independent variables individually).

**Categorical variable**							
**Variable**	**Levels of variable**	**Mean *ADJS***	**Std. deviation**	***t (df)***	***p*-value**	
*GHLDY*	General holiday	28.14	2.62	10.4 (88)	0.000	
	Non-holiday	21.66	3.14			
*EDUCTN*	Closed	25.06	3.86	16.9 (33)	0.000	
	Opened	16.20	0.76			
*FORCE*	Not in action	23.09	4.22	-4.8 (88)	0.000	
	In action	27.36	3.01			
*RELIGN*	Restricted	28.30	3.00	5.2 (88)	0.000	
	Permitted	23.30	4.02			
*MALL*	Closed	27.95	2.89	6.7 (88)	0.000	
	Opened	22.64	3.85			
*GRMT*	Closed	26.34	1.72	2.2 (88)	0.029	
	Opened	23.93	4.72			
*RMDN*	Days not in Ramadan	22.99	3.49	-13.5 (65)	0.000	
	Days in Ramadan	30.36	1.49			
**Continuous variable**							
**Variable**		**Correlation coefficient (*r)***				***p*-value**	
*PC*		0.407				0.000	
*NEWS*		0.421				0.000	

**Table 3:** Results of the developed multivariate multiple linear regression model.

Independent Variables	Coef. (*B*)	95.0% Confidence Interval for *B*		*p*-value	*VIF*
		Lower bound	Upper bound		
Constant	26.247	25.3	27.1	0.000	
*GHLDY*	-3.858	-4.9	-2.7	0.000	1.058
*EDUCTN*	-6.188	-8.0	-4.3	0.000	1.543
*RMDN*	4.118	2.7	5.4	0.000	1.486

Model Statistics

**F-test result:** *F*(3, 86)= 95.739, *p*-value= 0.000, *R*^2^ = 0.770.

ADJS=26.24−3.858GHLDY−6.188EDUCTN+4.118RMDN Eq (ii)

Here, *ADJS*= Average daily journey speed, *GHLDY*= Declaration of general holiday (0= general holiday, 1= non-holiday), *EDUCTN*= Closure of educational institutions (0= closed, 1= opened), and *RMDN*= Month of Ramadan (0= days not in Ramadan, 1= days in Ramadan).

### Declaration of general holiday (*GHLDY*)

The Government of Bangladesh declared general holidays from March 26, 2020 to May 30, 2020, [13, 17, 36, 37] while all types of public and private administrative, commercial, and industrial activities except emergency remained closed. During this period, the movement of public transport was also strictly controlled [17, 48–50]. **Fig 3** shows that the declaration of general holidays drastically increased the *ADJS* in Dhaka and the reopening of activities in June gradually decreased the *ADJS*. General holiday had massive statistically significant (*p*-value<0.01) impact on *ADJS* also evidenced from univariate analysis. *ADJS* increased from 21 km/hr to around 28 km/hr after the announcement of general holiday (**Table 2**). This strategy was also significant (*p*-value< 0.05) and had a negative coefficient (*B*) in the model (**Table 3**). The model showed *ADJS* decreased 3.099 km/hr if it was not a general holiday, holding all the other variables constant. Hence, the declaration of general holiday increased the *ADJS*, by keeping a large portion of vehicles off the road, and consequently helped to limit the spread of COVID-19. According to the Dhaka Structure Plan (2016-2035), almost 18% and 11.5% of trips in Dhaka are generated for “*to work*” and “*non home-based business (NHBB)*” purpose, respectively. In addition, 45% trips are for “*to home*” purpose where “*work to home*” purpose contains a substantial share [51]. Furthermore, about 49% of the “*home to work*” trips are made by motorized vehicles and 29% by rickshaw [52]. For this containment strategy, a large portion of the trips were not generated for “*to work*”, “*NHBB*” and “*work to home*” purpose, and consequently kept fewer vehicles on the roads and limited the viral transmission. “*Work from home*” approach might be adopted for the suitable jobs which would help to reduce the work trips during pandemic and minimize the considerable effects on economic and other sectors.

### Closure of educational institutions (*EDUCTN*)

The Ministry of Education closed all educational institutions across Bangladesh on March 18, 2020 in an effort to minimize the spread of COVID-19 [38, 39]. The institutions are not physically reopened yet [40]. After implementing this strategy, a sharp increase happened in the *ADJS* (**Fig 3)**. The univariate analysis showed, *ADJS* significantly increased from 16.2 km/hr to 25.1 km/hr (**Table 2**) which was also significant in the multivariate model (*p*-value<0.01) (**Table 3**). When educational institutions remained open, the *ADJS* decreased 6.149 km/hr, remaining other variables constant (**Table 3**). In Dhaka, 13% trips are generated for educational purpose and a major share of “*to home*” purpose trips are actually from “*educational institution to home*” [51]. Motorized vehicles are used for 29% of the educational trips and rickshaws are used for 41% of the trips [52]. Due to closure of educational institutions, lesser educational trips were made and ultimately led to reduce the traffic volume. Due to the closure of educational institutions, extensive interruption occurred on the students’ life [53]. To minimize the impact, online classes and classes through television could be attempted as alternatives.

### Deployment of force (*FORCE*)

Bangladesh government deployed the civil administrations and armed force on March 24, 2020 nationwide to ensure social distancing and strengthen the COVID-19 preventive measures [17, 41]. Deployment of force had significantly increased *ADJS* from around 23.1 km/hr to 27.3 km/hr in Dhaka according to the univariate analysis (*p*-value<0.01) (**Fig 3** and **Table 2**). However, this factor was not statistically significant in the multivariate model **(Table 3**). The civil administrations and armed force took steps to confine people inside home except for emergency purposes. They patrolled the whole city and checked the purpose of visit of the people who came outside from home [54]. There are also the evidences of actions, by the forces deployed, like physical harassment of the people who came outside without emergency purpose [55]. Several actions became viral on social media. These incidents increased fear among the people and discouraged people to go outside without any genuine purpose, and consequently helped to control vehicular traffic.

### Restriction on religious gathering (*RELIGN*)

Restriction on all type of religious gatherings was imposed urging people to perform their prayers and other religious rituals in their own houses to prevent the spread of COVID-19 by the government on April 6, 2020 [43]. This restriction was limitedly eased on May 7, 2020 by allowing people to pray in mosques [42]. *ADJS* increased significantly when restriction imposed on religious gathering according to the univariate analysis (*p*-value<0.01) (**Table 2**). However, this value is not statistically significant in the multivariate model. Therefore, this strategy did not have much impact on the *ADJS* cum control the traffic volume on the roads. However, this strategy was intended to ensure social distancing among the people rather preventing communal transmission through vehicular travel.

### Closure of market and shopping mall (*MALL*)

The Government of Bangladesh closed all markets and malls except the grocers’ shop, shops of daily essential commodities, and medicine shops across Bangladesh on March 26, 2020 [13]. However, from May 10, 2020, the government decided to allow all markets, shops, and shopping malls to remain open till 4:00 pm [44]. After reopening of them, *ADJS* slightly decreased, which means more vehicular traffic were present on the road (**Fig 3**). Result of univariate analysis also supports this result (**Table 2**), despite not significant, in multivariate model (**Table 3**). In Dhaka, trips generate for the shopping purpose is considerably much lower compared to the work trips and educational trips [51]. During the month of Ramadan, the share of shopping trips increase considerably due to doing Eid shopping. Despite the government allowed to open markets and malls, a large number of main markets and malls of Dhaka, including Bashundhara City Shopping Mall, New Market, Jamuna Future Park, Bongo Bazar remained closed by the market authorities [45]. If those markets and malls were reopened, *ADJS* might be decreased more. In addition, going to the shopping mall during pandemic might seem unsafe to people. Online shopping and mobile banking system can play a critical role during pandemic situation to reduce shopping trips, keeping vehicle off the road, and continuing shopping activities safely.

### Closure of garments factories (*GRMT*)

All garments factories of the country were declared to be closed by the respective authority (BGMEA and BKMEA) from March 26, 2020 to April 4, 2020. After a day of working on April 5, all the garments factories were shut again until April 25, 2020 [13, 46]. **Fig 3** shows that after reopening garments factories, *ADJS* decreased marginally. However, univariate analysis result indicated that this change was statistically significant (*p*-value<0.05) (**Table 2**), despite not being the same in the multivariate model. There are around 4,500 garments factories available in Bangladesh where 3.6 million workers work. Around 40% factories are located in Dhaka and about 35% of the total workers are employed here [56]. Most of the workers are not financially well-off and generally live within the walking distance of the factories [57, 58]. This might be the reason for insignificant impact of garments reopening on *ADJS*.

### Month of Ramadan (*RMDN*)

The month of Ramadan started at April 25, 2020 and end at May 24, 2020 [47]. During the month of Ramadan, *ADJS* increased drastically which means fewer vehicles were present on the road (**Fig 3**). This relationship was also statistically significant (*p*-value<0.01) both in univariate analysis (**Table 2**) and multivariate model (**Table 3**). According to the developed model, *ADJS* increased 4.524 km/hr during the month of Ramadan, keeping all other variables constant (**Table 3**). Around 90% people of Bangladesh are Muslim and most of them fast during the Ramadan. People who fast might be reluctant to go outside in the pandemic situation when most of the activities were not running. This could be a possible reason behind such increase in *ADJS* during Ramadan.

### Public concern and interest about COVID-19 (*PC*)

Public concern and interest about COVID-19 was found higher from the mid-March to the end of April (**Fig 4).** It was the highest during the mid-April and rapidly decreased from the beginning of May. **Fig 4** illustrates that the *ADJS* did not enormously depend on *PC*. Though univariate analysis confirmed a moderate significant positive correlation between them (*r*=0.41; *p*-value<0.01) (**Table 2**), *PC* was not found to be significant in the multivariate model (*p*-value>0.05) indicating *PC* did not have substantial impact on *ADJS*. Humans naturally are more attracted by new topics. Hence, after one and half months, citizens in Dhaka either felt indifferent regarding it or lost their interest on it [59, 60]. Thereby, people were more inquisitive at the beginning of the pandemic when fewer were infected. Hence, perhaps people felt that it was less necessary to stay home.

### Newspaper coverage on COVID-19 (*NEWS*)

**Fig 5** demonstrated a moderate positive relationship between *ADJS* and *NEWS* which was found significant in univariate analysis (*r*=0.42; *p*-value<0.01) (**Table 2**). However, like *PC*, this variable was also insignificant in multivariate model indicating number of news published per day did not have any substantial impact on controlling traffic volume. Though number of news published per day was not greatly varied over time, a small negative slope might be observed from the **Fig 5**. However, *ADJS* showed fluctuation over the period which mostly occurred for the implementation of different containment strategies and the month of Ramadan.

## Conclusion

To prevent the transmission of virus, it is exigent to restrict the movement of people, which can be elicited through the reduction of vehicular traffic on roads. This study investigated the effect of various containment strategies, impact of the month of Ramadan, public concern and interest, as well as media coverage on the vehicular movement of Dhaka, Bangladesh during the COVID-19 pandemic situation. This study considered the journey speed to understand the traffic volume scenario. The results of the study showed that the *ADJS* cum traffic condition of roads changes differently with the execution of different containment strategies. Strategies like declaration of general holiday and closure of educational institutions were the most effective strategies and able to increase the *ADJS* significantly which refers these strategies could extensively reduce the traffic volume on roads, and consequently lessening the risk of COVID-19 transmission. Therefore, in any emergency condition, government could apply these strategies to limit communal viral transmission by controlling human mobility. Despite the month of Ramadan is not a containment strategy, it was found that this event also had an extensive impact as a large portion of the people of Dhaka fast in this month. This finding suggested that it was easier to control movement during the month of Ramadan. On the other hand, closure of markets and shopping malls, restriction on religious gathering, and closure of garments factories could not bring significant change in traffic condition during pandemic. In addition, local cognition, e.g., people concern and interest as well as media coverage were not found significant indicating people responded more when government took strong steps (e.g., implementing containment strategies) to control pandemic situation. Eventually, the findings of this study would be helpful for decision-making to tackle the future pandemic situation in Dhaka as well as in other cities having similar characteristics.

## Supporting information

S1 Appendix Multiple linear regression model assumption checking document. (DOCX)
