# Supplementary material for: Effectiveness of containment strategies and local cognition to control vehicular traffic volume in Dhaka, Bangladesh during COVID-19 pandemic: Use of Google Map based real-time traffic data
Source: PLoS One. 2021 May 27;16(5):e0252228. doi: 10.1371/journal.pone.0252228 (PMC8158932; doi:10.1371/journal.pone.0252228)
Supplement: S1 Appendix — (DOCX) [file pone.0252228.s001.docx]

**S1 Appendix.**

**Assumption 1:** *Test of linearity* *between dependent variable and continuous independent variable:* A moderate linear relationship was found between dependent variable and continuous independent variable.


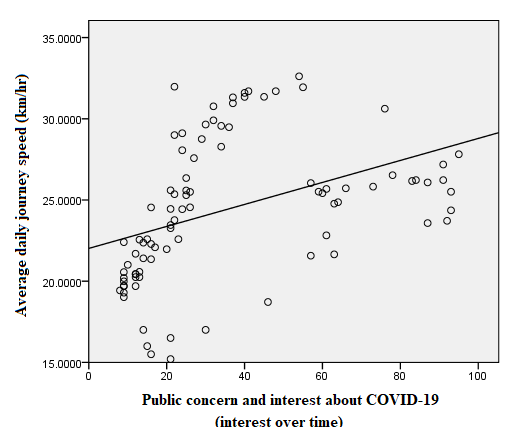

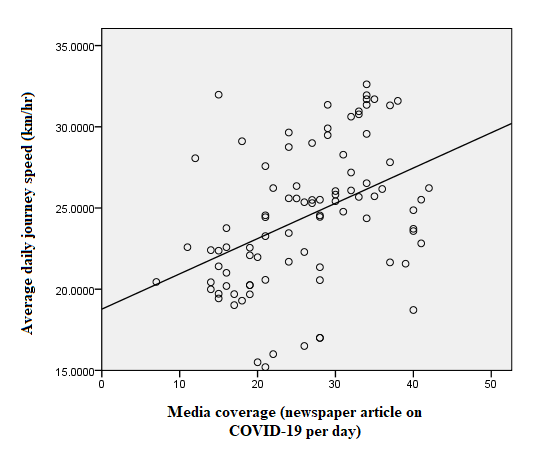


**Assumption 2:** *Test of homogeneity of variance:* No issue of heterogeneity was found.


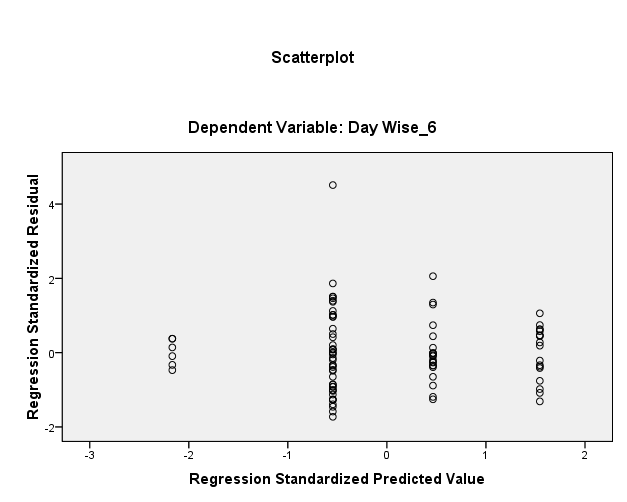


**Assumption 3:** *Test of normality of residuals:* Residuals were found normally distributed.


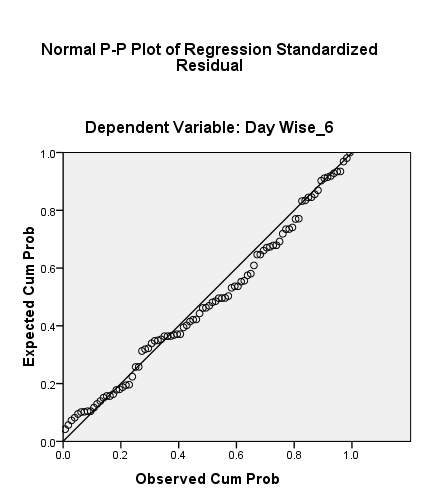


**Assumption 4:** *Test of multicollinearity:* From the model results, the highest VIF value was found 1.543 which is less than 5. Therefore, there is no issue of multicollinearity in the model.

**Assumption 5:** *Presence of no outlier:* No outlier was found in the data.

**Assumption 6:** *Presence of no auto-correlation*: For the developed model, Durbin-Watson value was found 1.28. According to the rule of thumb, values of Durbin-Watson between 1.5 < d < 2.5 show that there is no auto-correlation in the data. As Durbin-Watson value was found slightly lower than 1.5, it can be said that there are slightly first-order autocorrelation presence in the data. However, as here, sample size is 90 and numbers of independent variables in the model are three, therefore, this slightly first-order autocorrelation in the data could be overlooked due to small sample size and fewer numbers of independent variables.
